# Group membership and racial bias modulate the temporal estimation of in-group/out-group body movements

**DOI:** 10.1007/s00221-018-5313-4

**Published:** 2018-06-18

**Authors:** Valentina Cazzato, S. Makris, J. C. Flavell, Carmelo Mario Vicario

**Affiliations:** 1 Division of Psychology, University of Bradford, Bradford, UK; 2 Department of Psychology, Edge Hill University, Ormskirk, Liverpool, UK; 3 School of Optometry and Vision Science, University of Bradford, Bradford, UK; 4 Department of Psychology, University of York, York, UK; 5 Dipartimento di Scienze Cognitive, Psicologiche, Pedagogiche e degli studi culturali, Università di Messina, Messina, Italy; 6 Leibniz Research Centre for Working Environment and Human Factors, Dortmund, Germany; 7 University Medical Hospital Bergmannsheil, Bochum, Germany; 8 Present Address: School of Natural Science and Psychology, Liverpool John Moores University, Liverpool, UK; 9 Scienze Cognitive della Formazione e degli Studi Culturali, University of Messina, Messina, Italy

**Keywords:** Temporal estimation, In-group, Implicit embodiment, Action simulation, Race-IAT

## Abstract

Social group categorization has been mainly studied in relation to ownership manipulations involving highly-salient multisensory cues. Here, we propose a novel paradigm that can implicitly activate the embodiment process in the presence of group affiliation information, whilst participants complete a task irrelevant to social categorization. Ethnically White participants watched videos of White- and Black-skinned models writing a proverb. The writing was interrupted 7, 4 or 1 s before completion. Participants were tasked with estimating the residual duration following interruption. A video showing only hand kinematic traces acted as a control condition. Residual duration estimates for out-group and control videos were significantly lower than those for in-group videos only for the longest duration. Moreover, stronger implicit racial bias was negatively correlated to estimates of residual duration for out-group videos. The underestimation bias for the out-group condition might be mediated by implicit embodiment, affective and attentional processes, and finalized to a rapid out-group categorization.

## Introduction

There is mounting research evidence showing that the human body, and more specifically the human brain, are closely tied to the processing of both social and emotional information (Niedenthal et al. 2005). In that sense, researchers have proposed the notion of the embodied mind, suggesting that the processing of any mental content, such as perception and emotion, involves internal mental representations (Barsalou et al. 2003; Smith and Semin 2004). The main idea underlying all theories of embodied cognition is that cognitive operations rely heavily on the brain’s modality-specific networks and on actual bodily states (Niedenthal et al. 2005). For example, empathy and understanding of another person’s emotional state requires mental recreation of that person’s feelings in ourselves. Similarly, observing an action sequence and predicting its outcome require the activation of the observer’s motor cortex and the use of internal motor representations for simulating the observed action (Wilson and Knoblich 2005; Keysers and Gazzola 2009; Avenanti and Urgesi 2011; Friston et al. 2011). The aforementioned phenomenon is known as sensorimotor mirroring and its underlying theory has been enormously influential in the cognitive sciences and beyond, as it has provided the ground of a unified model for sharing representations and social cognitive functions from intention understanding to actions and empathy (Niedenthal et al. 2005).

More recently, research has shown that social group categorization triggered by skin color can modulate sensorimotor mirroring during observation of neutral actions; for example, in culture-specific gestures (Molnar-Szakacs et al. 2007; Désy and Théoret 2007; Gutsell and Inzlicht 2010) as well as in empathic sensorimotor and affective mapping of observed painful stimulation (Avenanti et al. 2010; Azevedo et al. 2013; Liew et al. 2011). There is also evidence that the perception of ethnic-related features modulates brain activity associated with action observation and imitation (Earls et al. 2013; Losin et al. 2012) and observation of one’s ethnic group while performing an action (i.e., a Chinese or a Caucasian actor performing a familiar or non-familiar gesture) can modulate the so-called ‘mirror neuron system’ during intention understanding (Liew et al. 2011). This is in line with Maister et al.’s (2015) suggestion that social group categorization and group membership can have a strong impact on the extent to which we resonate with others. Soliman et al. (2013) found that interdependent Americans (a group stressing relatedness and harmony with their in-groups) perceive American confederates (an in-group) as spatially and temporally closer, than independent Americans (a group stressing the uniqueness of the individual) or Arab confederates.

It has also been shown that the so-called in-group bias may strongly affect bodily illusions such as the ‘Rubber Hand Illusion’ (Maister et al. 2013; Peck et al. 2013). Interestingly, people with high-levels of prejudice show reduced embodied resonance with other-races compared to their own race. This in turn correlates with participants’ negative implicit racial biases (Gutsell and Inzlicht 2010; Avenanti et al. 2010; Azevedo et al. 2013; Sacheli et al. 2015). Moreover, the phenomenon of embodiment is associated with the formation of prejudice and stereotypes (Groom et al. 2009), and is considered an important sensorimotor predictor for empathy (e.g., Avenanti et al. 2005). Importantly, empathy itself has been considered relevant for explaining in-group/out-group dynamics, given the evidence that people routinely fail to empathize with members of different social or cultural groups (Cikara et al. 2014).

Recent evidence indicates that the subjective temporal experience of an *emotional* event is longer than that of a *neutral* event (Angrilli et al. 1997; Droit-Volet et al. 2004) and that embodiment of stimulus characteristics may be required for such emotionality influences (Effron et al. 2006). The embodiment of facial expressions can affect autonomic arousal (Droit-Volet et al. 2004) so mimicry of any emotional facial expressions might activate arousal–valence-based mechanisms that seem to be responsible for temporal mis-estimation effects (see Angrilli et al. 1997).

The literature outlined so far highlights the importance of emotional embodiment in social group categorization, group membership and time perception. Yet, our knowledge of cognitive mechanisms involved in group membership and categorization is limited, as it has been mainly focused on the manipulations of bodily illusion-inducing paradigms. Though extremely relevant for the field, these paradigms have been criticized for involving highly salient multisensory (i.e., tactile, visual, motor) cues. Such cues might be strongly predictive of body ownership and for this reason may blur the effect of social attitudes on body resonance with others, as suggested by some scholar (e.g., Maister et al. 2013, 2015; Peck et al. 2013). Similar concerns might also apply to virtual environmental experiments where the participant is explicitly asked to embody an avatar of different ethnicities (e.g., Groom et al. 2009). To exceed such limits/concerns, we decided to adopt a task of temporal estimation of body movements, where participants have to estimate the residual duration of Black-skinned (out-group) or White-skinned (in-group) hand-writing movements that are occluded several seconds before completion. Implicit activation of the embodiment process via action simulation (Springer et al. 2013, i.e. embodying the writing movement) is necessary for completion of this task (Nather et al. 2011; Avanzino et al. 2013; Vicario et al. 2017). A novel aspect of our procedure is that of prompting the embodiment process in the presence of social group categorization/membership cues whilst participants focus on information irrelevant to social categorization (i.e., the duration of the displayed actions). In other words, social group categorization/membership was experienced as background information rather than being the participants’ goal as in some previous studies (e.g., Maister et al. 2013; Peck et al. 2013).

It is important to note that using a test of three domains of executive function (response inhibition, updating, and shifting), Ito et al. (2015) reported that executive function and estimates of automatic processes can predict implicit racial bias. In keeping with this study (Ito et al. 2015), with our paradigm on time processing, we extended the investigation on the linking executive function/implicit racial bias to two unexplored domains of the executive function which are considered essential for time estimation: attention (e.g., Meck and Benson 2002; Lewis and Miall 2006; Vicario 2013) and working memory (e.g., Lewis and Miall 2006; Buhusi and Meck 2005; Matthews and Meck 2016). Importantly, working memory has been linked to processes of embodiment (Wilson 2002).

In addition, to more specifically evaluate the contribution of group membership and group categorization to embodiment, during the current timing task, participants completed a computerized version of the race-implicit association test (race-IAT, Greenwald et al. 1998). Rather than exploring the extent to which participants explicitly considered Black-skinned people to be out-group members, here, we use an implicit race-IAT task because controlled, belief-based processes are more effectively implemented in deliberative responses such as self-report questionnaires (Amodio et al. 2003). Amodio et al. (2003) showed that racial bias is more readily observed at an implicit rather than at an explicit level, possibly because explicit measures may offer a greater chance of regulating the expression of bias (Greenwald et al. 1998; Dunham et al. 2008; Eberhardt 2005).

Given the link between embodiment to social group representation (e.g., Maister et al. 2013; Peck et al. 2013), as well as evidence that the temporal estimation of body movements is influenced by embodiment (Nather et al. 2011; Avanzino et al. 2013; Vicario et al. 2017), we predict that participants will differently estimate the completion time of in-group and out-group writing movements. Negative stimuli can lead to temporal underestimation (e.g., Angrilli et al. 1997) so tendencies to show negative attitudes towards an out-group (e.g., Nawata and Yamaguchi 2014) should lead to temporal underestimation for out-group movements (relative to in-group). We also expected to detect a negative correlation between IAT scores, which provide a measure of implicit racial bias of participants, and the time estimation performance. The higher the implicit racial bias, the lower the perceived duration of the out-group movement.

## Methods

### Participants

Sample size was previously calculated using a freely-available G*Power software (G*Power 3.1.9; Faul et al. 2007), estimating a sample of 22 participants as adequate for a design with 95% power to detect a moderate effect size between the variables (*f* = 0.25). However, because of the possibility that a small number of participants would produce unreliable temporal estimation data a total of 27 White (self-selected as ‘British, Irish or other White Background’) right-handed (determined by Standard Handedness Inventory, Briggs and Nebes 1975) students from the University of Bradford participated (age (mean ± SD) = 22.11 ± 2.76 years, 16 were female) in the study. Participants were naïve to the purposes of the experiment. Information about the experimental hypothesis was provided only after the experimental tests were completed. All participants reported normal or corrected-to-normal vision were in good health, free of psychotropic or vasoactive medication, and had no history of psychiatric or neurological disease. Participants gave their written informed consent and procedures were approved by the University of Bradford’s local ethics committee and were in accordance with the ethical standards of the 1964 Declaration of Helsinki.

### Temporal Estimation Task

The Temporal Estimation Task was a modified version of the paradigm originally used by Avanzino et al. (2013) and by Martino et al. (2015) (see Avanzino et al. 2016, for a review). The current task was realized using E-Prime (v2.0, Psychology Software Tools, Inc., Pittsburgh, PA, USA) and consisted of four stimulus videos. In all of them, the camera view was aimed from the point of view of right-handed male actors whilst they wrote “The early bird catches the worm” using a black pen on an A4-size white paper positioned on a table. In one video, the actor was Black-skinned (out-group) and in another the actor was White-skinned (in-group). All participants were White skinned, so the White-skinned video was always the in-group and the Black-skinned video was always the out-group. The other two videos acted as a control condition (no-group). In these (control) no-group videos, a single tracking marker was placed on the back of the actor’s writing hand between the second and third metacarpals and half-way up their length. Passive markers were also placed on the corners of the paper to record its boundary. When rendering the video, the paper edges were shown as blue lines and the marker on the back of the participants’ hand as a black sphere. For rendering, the virtual camera was positioned and calibrated to provide a near identical perspective as the ‘real’ videos. This way, participants could only see ‘hand-writing kinematics’ trace with no racial cues available to them. To generate the control videos, the 3D positions of the hand and paper during writing were tracked (at 100 Hz) using four Bonita cameras (Oxford Metrics, Oxford, UK) and Vicon Nexus 1.8.5 (Oxford Metrics, Oxford, UK). Trajectories were filtered with a second-order 10-Hz low-pass Butterworth filter before exporting to Matlab (R2014a The MathWorks Inc., MA, USA) where they were rendered into an MPEG files. All actors were trained to complete the writing movements in the same time (~ 13 s) and over approximately the same distance.

**Fig. 1:**
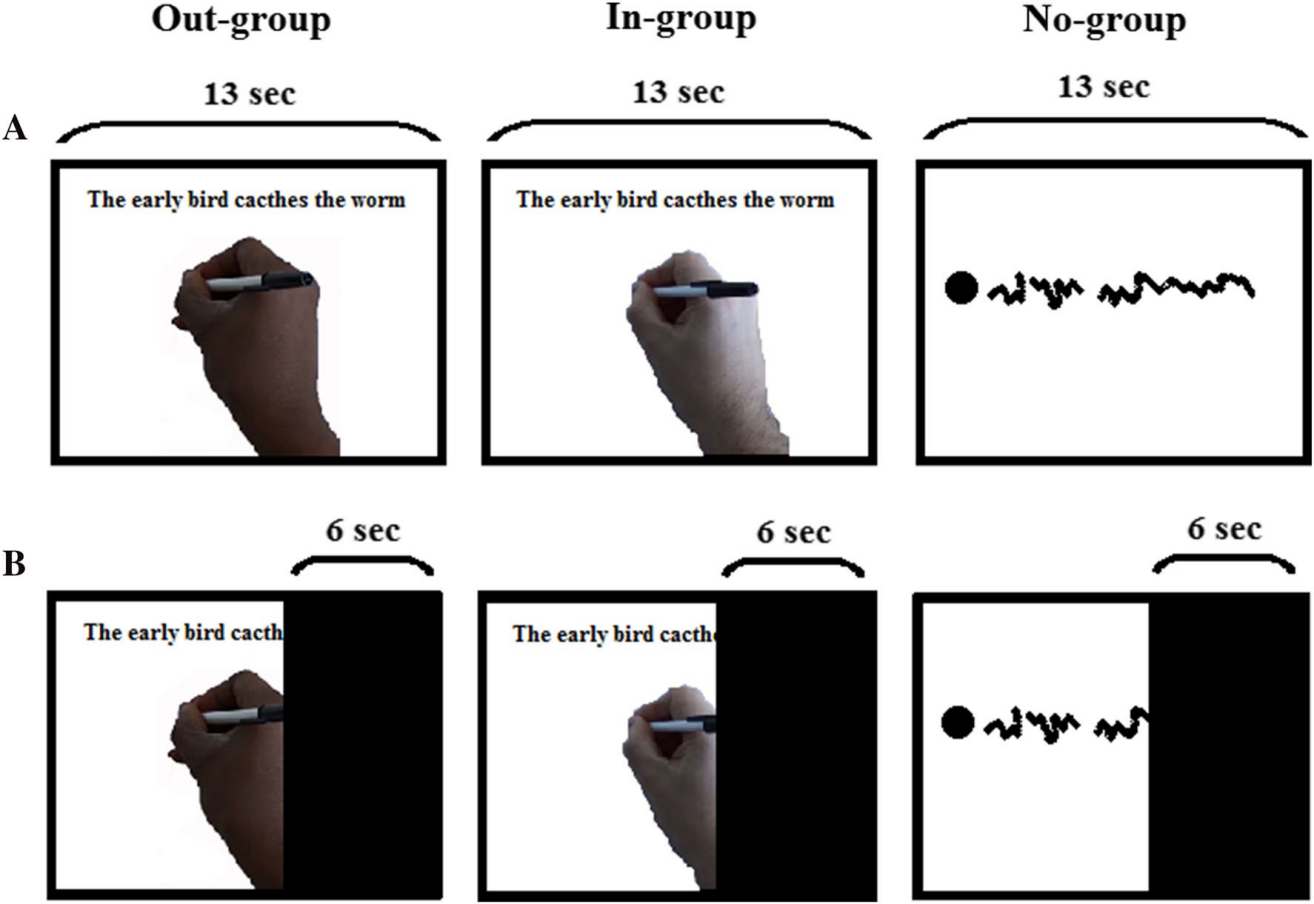
A schematic depiction of the Temporal Estimation Task. **a** In familiarization trials, participants were presented with videos of writing a proverb. Writing was conducted by an out-group (Black-skinned) actor, an in-group (White-skinned) actor, and a control showing only a hand kinematics trace (No-group). **b** In experimental trials, the videos were interrupted 7, 4 or 1 s before completion. For simplicity, only the 7-s interruption is depicted

During the experimental sessions, participants sat in a darkened room 57 cm away from a 15.6-inch LCD monitor (1024 × 768 pixels, 60 Hz). Participants used a keyboard in front of them to input responses. At the start of the experiment, each participant was first shown each video in full (in a random order) without receiving instructions for the upcoming task. In the experimental trials, participants saw the same videos as before but this time the videos were interrupted by a gray screen 7, 4 or 1 s before completion. The participants’ task was to estimate the residual duration by pressing the space bar when they believed the writing movement would have been completed. The gray interruption screen remained until participants made a response. Figure 1 shows a schematic depiction of the Temporal Estimation Task.

Experimental trials were blocked by a group (in-group, out-group, or no-group). There were 10 repetitions of each group video at every interruption time to create 30 trials per block and a total of 90 experimental trials. Note that the no-group condition contained five repetitions of the Black actor’s video and five repetitions of the White actor’s video. Block order was randomized between participants. No feedback on estimation accuracy was provided. Different intervals were included to promote attention and reduce the boredom of task repetition (Haager et al. 2016), that can negatively affect cognitive performance (see Fisher 1993; Tze et al. 2016 for review).

### Race-IAT

The race-IAT (race implicit association test) was created using E-Prime (v2.0 Professional, Psychology Software Tools, Inc., Pittsburgh, PA, USA). Participants were shown single words or images of Black or White faces one at a time in the center of the screen and were asked to respond as quickly and as accurately as possible to them. Participants made responses by pressing a left key (E) or right key (I) on a computer keyboard with the index finger of their left or right hand, respectively. The IAT was administered in seven total blocks, consisting of both congruent and incongruent condition blocks (blocks 3, 4, 6, and 7) and familiarization blocks (blocks 1, 2, and 5) (Greenwald et al. 2003; Cattaneo et al. 2011; Crescentini et al. 2014; Cazzato et al. 2017). Before the first presentation of the race-IAT, participants were asked to carefully read a list of all the single words that would be presented later. In the first block, 12 images of White faces and 12 images of Black faces were presented and had to be classified as being either ‘Bad’ (left key) or ‘Good’ (right key). Each image was presented only once, for a total of 24 trials. The second block also consisted of 24 trials in which Bad-related (requiring a left key response) and Good-related (requiring a right key response) words were presented. In the third block (24 practice trials) and in the fourth block (48 experimental trials), both Black and White faces, and Good and Bad words were randomly presented and participants were instructed to press the left key for Bad-related words and for images of Black faces, and the right key for Good-related words and for images of White faces. This was the congruent-stereotype condition. In the fifth block (24 experimental trials), response key assignments were reversed in relation to the categorization involving images of Black faces (right key) and White faces (left key). Finally, in the sixth block (24 practice trials) and in the seventh block (48 experimental trials), both Black and White faces, and Good- and Bad-related words were randomly presented and participants who were required to press the left key for images of Black faces and for Good words and the right key for images of White faces and for Bad words. This was the incongruent-stereotype condition. Participants who hold implicit negative bias towards Black people are typically faster and more accurate in such congruent-stereotype blocks than they are in such incongruent-stereotype blocks, thus demonstrating an automatic association between Black and Bad categories and between White and Good categories (Greenwald et al. 2003). Stimuli were randomly ordered within each block. Each word/image remained on the screen until the participant gave a correct response in each trial. If participants made a mistake on a trial, a red “X” appeared below the word stimulus to prompt the participant to make a correct response. Following a correct response, the next stimulus appeared after 500 ms, during which only the category labels were visible on the screen. The race-IAT took ~ 8 min.

### Statistical analysis

Three participants were excluded from all analyses because they misunderstood the Temporal Estimation Task and performed it in a reversed way (i.e., the lower the temporal distance between the interruption time and the movement completion, the higher the estimated residual duration). Two participants were also removed because of outlier scores (± 3 SD of the group) in the 1-s interruption interval of the Temporal Estimation Task. Therefore, the final ANOVA included 22 participants, as one temporal interval condition was missing. On the other hand, 23 participants were available for the correlation analysis that included the race-IAT score and the mean score of the three intervals provide for the type of videos. All data were analyzed using STATISTICA 8.0 (StatSoft Inc, Tulsa, OK, USA).

In the Temporal Estimation Task, responses to the no-group videos were averaged together to provide a single control variable (i.e. no separation of White and Black actors in this anonymized condition). We also calculated the coefficient of variation (standard deviation/mean of the estimated residual duration) which represents a trial-by-trial measure of performance variability in temporal estimation (Vicario et al. 2011a, b, 2013). Temporal estimation and the coefficient of variation scores were submitted to 3 × 3 repeated measures ANOVAs with the factors standard residual duration (7, 4 and 1 s) and videos (in-group, out-group and no-group). Significant interactions were explored using Bonferroni corrected *t* tests. Effect sizes were estimated using the partial eta square measure (*ηp*^2^). Pearson’s correlation coefficients were computed to explore the relationship between the temporal estimation score and the degree of individual racial prejudices as measured by the race-IAT. Significance was assessed at *α* = 0.05 in all tests.

## Results

### Temporal Estimation Task

**Fig. 2:**
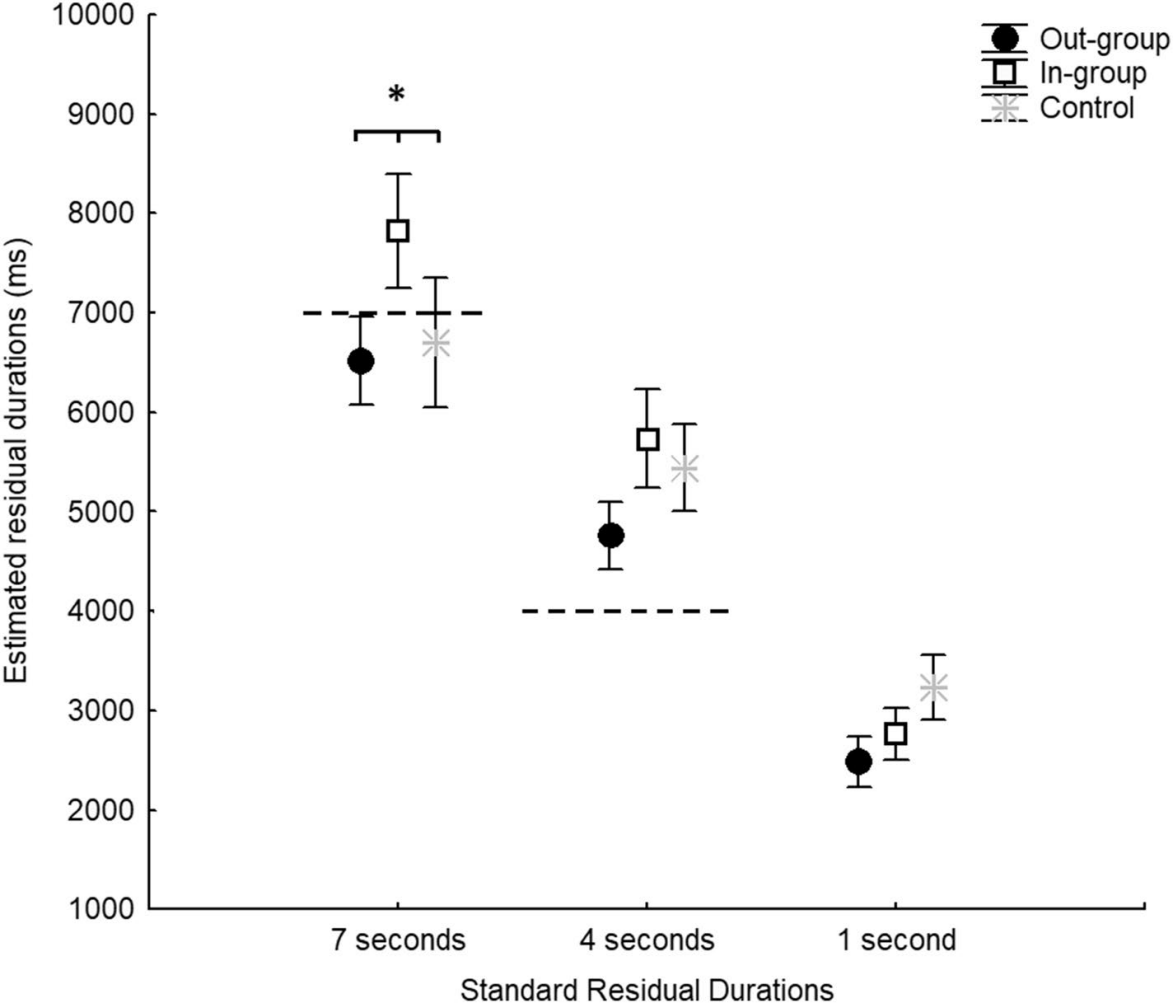
Mean and SEM of estimated temporal duration for residual durations in each movement group (in-, out- and no-group). Dashed lines indicated the allocation of residual duration regarding the *Y* axis. Significant differences are indicated by “asterisk”

As expected, ANOVA indicated a significant main effect of standard residual duration [*F*_(2,42)_ = 140.0, *p* < 0.001, *ηp*^2^ = 0.869]. It also indicated a significant interaction of residual duration × video [*F*_(4,84)_ = 3.228, *p* = 0.016, *ηp*^*2*^ = 0.133]. Post hoc analysis revealed that at the longest residual duration (7 s), participants’ estimates for in-group videos (*M* = 7825.6 ms, SE = 573.6) were significantly longer than for out-group (*M* = 6520.9, *SE* = 445.1, *p* = 0.003) and neutral (*M* = 6704.2, SE = 653.5, *p* = 0.026) videos. In contrast, we did not detect a significant difference between out-group and no-group videos (*p* = 1.000). No significant difference was reported at the 4-s residual duration (*p* > 0.116). Finally, we did not detect a significant difference at the 1-s residual duration (*p* > 0.811). No significant difference was reported for the video factor [*F*_(2,42)_ = 1.814, *p* = 0.175, *ηp*^2^ = 0.079]. Data are plotted in Fig. 2.

We also compared the difference between participants’ temporal estimations and the residual durations for each video and standard residual duration. Single sample *t* tests revealed significant overestimations for all the three videos at the 4- and 1-s standard residual durations only (*p* ≤ 0.010). For the 7 s standard residual duration, we found a no significant overestimation trend for the in-group (*p* = 0.089). In contrast no significant results were reported for the out-group (*p* = 0.670) and the no-group video (*p* ≥ 0.675).

The ANOVA on coefficient of variation indicated a significant main effect of residual duration [*F*_(2,46)_ = 10.0, *p* < 0.001, *ηp*^2^ = 0.303], but no other significant effects (*p* > 0.170).

### Race-IAT

Race-IAT scores were analyzed using Greenwald et al.’s (2003) scoring algorithm, where *D*-scores greater than zero suggest the presence of the implicit racial bias. A one sample *t* test was used to compare the group mean *D*-score (*M* ± MSE = 0.437 ± 0.27) to zero (where zero refers to the absence of any response bias). Participants showed a significant racial bias, indicating that they were more likely to associate Black faces to the Bad-related category and White faces to the Good-related category than vice versa [*t*(22) = 7.75, *p* < 0.001].

### Correlations

**Fig. 3:**
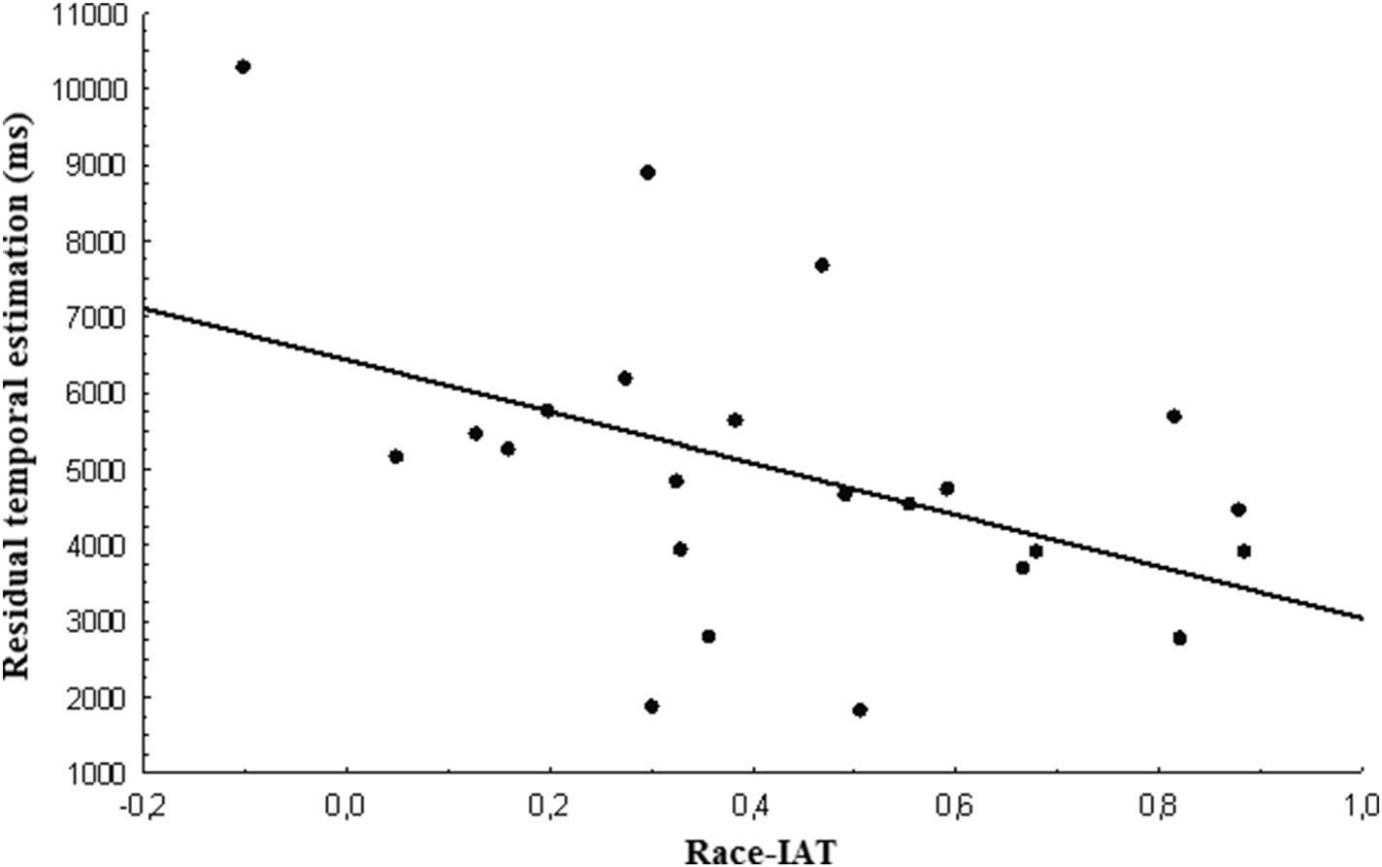
Correlation of mean estimated residual durations for out-group movements (Black actor) vs. race-IAT scores

Correlation analyses were conducted with race-IAT vs. the mean of residual temporal estimation at the three interruption times for each video. Note that this involved 23 rather than 24 participants as we were not able to collect this information for one participant (see “Methods”). There was a significant negative correlation between the race-IAT scores and the residual temporal estimation for the out-group (*r* = − 0.457, *p* = 0.028). Thus, the stronger implicit racial bias the lower the overall residual temporal estimation for the out-group (see Fig. 3). There were no significant correlations in either of in-group or no-group videos (*p* > 0.160). We also performed a further analysis between the race-IAT score and the normalized timing score obtained by applying the following formula: (Black condition − White condition)/control condition. We detected a significant negative correlation (*r* = − 0.358, *p* = 0.046).

## Discussion

In the present work, we aimed to test theoretical models (e.g., Glenberg 2010; Maister et al. 2015) and evidence (e.g., Avenanti et al. 2010; Maister et al. 2013; Soliman et al. 2013; Groom et al. 2009) suggesting that social group categorization and group membership are mediated by processes of embodiment. To this end, we employed a task of temporal estimation of Black (out-group) and White (in-group) hand-writing movements. This task prompts implicit activation of the embodiment process via action simulation (Springer et al. 2013), and investigates social group categorization/membership, whilst participants focus on information that is irrelevant to social categorization (i.e., the displayed actions). We tasked participants with estimating the residual duration of an interrupted video of hand-writing performed by an out-group actor, an in-group actor, or by a trace of hand-writing kinematics (no-group). Moreover, we investigated whether duration estimates were predictors for implicit racial bias as measured by the race-IAT.

Overall, we found a significant overestimation of the 4- and 1-s residual durations. This is in line with previous reports using different actions (e.g., Sparenberg et al. 2012), and might reflect a slower perception of time when predicting ongoing actions. The trend of reducing temporal error as residual duration increases is similar to that found in many prediction motion studies (e.g. Flavell et al. 2018). Specifically, responses of movement completion tend to occur less late and/or increasingly early as the residual duration increases. It thus seems possible that temporal estimation processes may be somewhat generalized.

In line with our initial prediction, we found that participants significantly underestimate residual duration of out-group and no-group movements compared to in-group movements, though only at the longest residual duration of 7 s. By contrast, there was no significant difference between the out-group and no-group estimated residual durations.

We also found a negative correlation between temporal estimation for the out-group video and the race-IAT scores. Thus, the stronger implicit racial bias the lower the overall temporal estimation associated to the out-group video. The absence of relationships between the timing performance for the no-group condition and the race-IAT score indicates that the implicit racial bias does not affect, per se, temporal estimation performance. Therefore, one might conclude that this mental construct has only a marginal effect on working memory and attention, which are supposed to be involved in the execution of the current timing task, as suggested by several theoretical and experimental investigations (e.g., Lewis and Miall 2006; Vicario et al. 2011a, b, 2012; Vicario and Martino 2010; Meck and Benson 2002; Buhusi and Meck 2005; Matthews and Meck 2016). However, this assumption remains speculative, as we did not include standard tests to measure such cognitive dimensions.

It has been suggested that the perception of movements performed by members of the same species may rely on processes of motor simulation or internal action (Ramnani and Miall 2004; Urgesi et al. 2010). More specifically, it has been found that action perception is inherently linked to motor representations and that subjective experience serves a critical role in the recognition and simulation of ongoing actions (Hecht et al. 2001; Casile and Giese 2006; Aglioti et al. 2008; Urgesi et al. 2012). The evidence of a significant difference only at the longest residual duration might depend on the degree of internal action simulation, which may have been higher (i.e., facilitated) for the 7-s standard residual duration, compared to the others residual durations. However, this hypothesis remains speculative, as we did not collect measures of motor simulation in relation to the residual durations. Despite this, several potential hypotheses can be provided to interpret the current result.

Theories of embodied cognition suggest that the ability to represent, simulate and manipulate information that sometimes is not even perceptually present can be accomplished through the activation of sensorimotor processes (Hostetter and Alibali 2008). For example, observing an action sequence and making predictions of its outcome would not only require the activation of the observer’s visual and motor systems, but also the use of internal sensorimotor representations for making accurate predictions (Hostetter and Alibali 2008; Prinz 1997). However, it has been demonstrated before that this parallel activation and switch between perceptual modalities comes with a cost (Pecher et al. 2004; Solomon and Barsalou 2001; Zwaan and Yaxle 2003). For example, it has been shown that in sensorimotor mirroring it is extremely difficult to process information by contradicting the contingencies between perception and action that exist in the physical environment (Hostetter and Alibali 2008; Schwartz and Black 1999). First, assuming that a process of embodiment, necessary for accurate temporal estimations of body actions, takes place only for the in-group, one might observe that this process is detrimental to performance. In fact, there is a larger temporal gap (i.e., error) between the residual duration and the estimated duration for the in-group than either the out-group or no-group. Therefore, contrary to our initial prediction, one might conclude that the process of embodiment worsens temporal estimation in the direction of over-estimation.

An alternative interpretation, assuming the tendency to overestimate ongoing actions is a normal process as suggested by some studies (e.g., Sparenberg et al. 2012), is that the temporal underestimation documented for the out-group is the sign of abnormal performance, which might reflect a phenomenon of *disembodiment* and/or reduced *action simulation*. This interpretation has been provided in keeping with the evidence of a similar timing performance for the out-group and the no-group, where indeed body in action information was not available. In line with this hypothesis, one might suggest that the process of *disembodiment* (and/or reduced *action simulation*) might reflect an implicit mechanism finalized to a rapid out-group categorization and mediated by implicit racism, as suggested by the results of the correlation analysis. Importantly, although we did not directly measure the ability to simulate actions in our participants, this interpretation fits with the study of Gutsell and Inzlicht (2010), which demonstrated a reduced mental simulation of actions during observation of out-group acts by studying the activity over the motor cortex of participants. Moreover, a recent study (Greven and Ramsey 2017) has documented that a cluster of the right Fusiform Body Area, a neural region implied in processing whole-person body representation (Downing and Peelen 2011) had a greater functional coupling with left temporo-parietal junction (a key region of the Theory of Mind network which plays a central role on social cognition, Schuwerk et al. 2017) when recalling positive and negative traits about in- and out-group members.

The emotional valence associated with the in-group and out-group videos might provide further relevant details to understand our results. Angrilli et al. (1997) found that when employing low-arousing pictures, the duration of unpleasant pictures was underestimated, while the duration of pleasant pictures was overestimated. One might then hypothesize that our finding of duration overestimation for the in-group video compared to the out-group and no-group videos (at 7 s standard residual duration) might reflect the positive experience prompted by in-group bias. Another suggestion is that the underestimation pattern associated with the out-group, compared to the in-group, might reflect the negative experience prompted by out-group bias. In line with this suggestion, which is in agreement with our initial hypothesis, the reported result might be the effect of a valence-driven bias, which can be grounded in processes of embodiment (Casasanto 2009). In the context of the current discussion on the emotional valence hypothesis, it is also important to mention the potential role of participants’ arousal, as it might have significantly affected the time clock speed (Droit-Volet et al. 2004, Droit-Volet 2013; Droit-Volet and Gil 2009; Droit-Volet and Meck 2007). Accordingly, one might speculate that the exposure to out-group stimuli might have caused an increment of the subjective arousal, which is known to speed-up the mental clock (Droit-Volet and Meck 2007), with the consequent underestimation bias.

Finally, another possible explanation for our results is that the underestimation bias reported for the out-group, compared to the in-group, might reflect higher attentional capturing of the out-group cue (i.e., the skin color). This is supported by previous reports documenting an attention bias of white people for black faces (e.g., Trawalter et al. 2008), and a temporal underestimation when stimuli to be timed use attentional resources (e.g., Angrilli et al. 1997; Droit-Volet and Meck 2007).

## Limitations

The current study presents some limitation that should be mentioned. First of all, we did not provide a control condition for the difference in the visual contrast between the Black-hand and the White-hand to the paper that was written. Hand contrast of the white background may have affected temporal estimation. However, the use of a white background has the advantage to be ecological, because non-white writing mediums are uncommon. Second, we did not provide a control task testing the effect of in-group/out-group cues on timing performance in the absence of body information. Third, we did not perform kinematic analysis to identify potential differences between the Black actor’s movements and the White actor’s movements. However, any potential differences are expected to be minor because both actors were trained to perform the task similarly.

## Conclusions

In the current study, we show that group membership prompted by skin color affects the residual temporal estimation of hand-writing movements. We found underestimation for out-group movements, which is also related to the implicit racial bias measure of our participants, as shown by the correlation analysis. Overall, our results suggest that a process of *disembodiment* (or *reduced action simulation*), probably mediated by the specific affective (i.e., valence related) and attentional/arousing information, might be automatically prompted by the exposure to out-group information.

In conclusion, our results are in line with models theorizing a role of embodiment in the representation and perception of group membership (e.g., Soliman et al. 2013; Maister et al. 2015) and in social cognition (e.g., Niedenthal et al. 2005) because these corroborate the suggestion that the process of embodiment is a central element in the psychological building of the self/other representation.
